# Supplementary material for: Role of intraoperative oliguria in risk stratification for postoperative acute kidney injury in patients undergoing colorectal surgery with an enhanced recovery protocol: A propensity score matching analysis
Source: PLoS One. 2020 Apr 17;15(4):e0231447. doi: 10.1371/journal.pone.0231447 (PMC7164643; doi:10.1371/journal.pone.0231447)
Supplement: S1 Table — (DOCX) [file pone.0231447.s001.docx]

**S1 Table.** ERAS protocol of Seoul St. Mary’s hospital in patients undergoing colorectal cancer surgery

| Period | Component | Content |
| --- | --- | --- |
| Preoperative | Preadmission patient education | At the outpatient department |
|  | Preoperative oral carbohydrate treatment | By 2 hours before surgery |
|  | Preoperative formula intake | Parenteral nutrient solution after mechanical bowel preparation |
|  | Thrombosis prophylaxis | Preoperative vascular surgery team consultation and  application of a pneumatic compression pump |
|  | Antibiotics prophylaxis | Administered at 30 minutes before incision |
| Intraoperative | Epidural anesthesia and postoperative analgesia | Using at least one day of PCA through epidural route |
|  | Body temperature preservation | Use of air warmer and trans-esophageal monitoring device |
|  | Restrictive fluid strategy | Zero-balance fluid therapy |
|  | PONV prophylaxis | Administered before the end of surgery |
| Postoperative | Effective pain control | Well controlled pain with NSAID only |
|  | Postoperative fluid balance | Daily total fluid level 500 mL or less |
|  | Stimulation of gut motility I | Laxatives used |
|  | Stimulation of gut motility II | Chewing gum used |
|  | Termination of urinary drainage | Withdrawal of Foley catheter before the third postoperative day |
|  | Drainage remove | Until the third postoperative day |
|  | Termination of intravenous fluid infusion | Until the third postoperative day |
|  | Mobilization on day of surgery | Postoperative movement outside the bed |
|  | Mobilization on postoperative day 1 | Over 4 hours |
|  | Mobilization on postoperative day 2 | Over 6 hours |
|  | Mobilization on postoperative day 3 | Over 6 hours |
|  | Energy intake on day of surgery, postoperatively | Intake 200 kcal or more |
|  | Energy intake on postoperative day 1 | Intake 500 kcal or more |

**Abbreviations:** ERAS, enhanced recovery after surgery; PCA, patient-controlled analgesia; PONV, postoperative nausea and vomiting; NSAID, non-steroidal anti-inflammatory drug.
