# Supplementary material for: Role of intraoperative oliguria in risk stratification for postoperative acute kidney injury in patients undergoing colorectal surgery with an enhanced recovery protocol: A propensity score matching analysis
Source: PLoS One. 2020 Apr 17;15(4):e0231447. doi: 10.1371/journal.pone.0231447 (PMC7164643; doi:10.1371/journal.pone.0231447)
Supplement: S2 Table — (DOCX) [file pone.0231447.s002.docx]

**S2 Table.** Definition of acute kidney injury according to Kidney Disease Improving Global Outcomes stage

| Stage | Serum creatinine | Urine output |
| --- | --- | --- |
| 1 | 1.5 – 1.9 × increase from baseline SCr within 7 days  OR  ≥ 0.3 mg/dL increase in SCr within 48 h | < 0.5 mL/kg/h for 6 h |
| 2 | 2.0 – 2.9 × increase from baseline SCr within 7 days | < 0.5 mL/kg/h for 12 h |
| 3 | ≥ 3 × increase from baseline SCr within 7 days  OR  Increase in serum creatinine to ≥ 4 mg/dL  OR  Initiation of renal replacement therapy | < 0.3 mL/kg/h for 24 h  OR  Anuria for 12 h |

**Abbreviation:** SCr, serum creatinine.
